# Gamma-Aminobutyrate Transaminase Protects against Lipid Overload-Triggered Cardiac Injury in Mice

**DOI:** 10.3390/ijms23042182

**Published:** 2022-02-16

**Authors:** Mengxiao Zhang, Huiting Zhong, Ting Cao, Yifan Huang, Xiaoyun Ji, Guo-Chang Fan, Tianqing Peng

**Affiliations:** 1 Institutes of Biology and Medical Sciences, Soochow University, Suzhou 215123, China; zmx1088@163.com (M.Z.); zht0905zht@163.com (H.Z.); tcao704@163.com (T.C.); yyzhyf@hotmail.com (Y.H.); 2 School of Pharmacy, Bengbu Medical College, Bengbu 233000, China; 3 Department of Pathology and Laboratory Medicine, Western University, London, ON N6A 5C1, Canada; xji86@uwo.ca; 4 Lawson Health Research Institute, London Health Sciences Centre, London, ON N6A 5W9, Canada; 5 Department of Pharmacology and Systems Physiology, University of Cincinnati College of Medicine, Cincinnati, OH 45267, USA; fangg@ucmail.uc.edu; 6 Department of Medicine, Western University, London, ON N6A 5W9, Canada; 7 VRLA6-140, 800 Commissioners Road, London, ON N6A 5W9, Canada

**Keywords:** lipid overload, ABAT, mitochondrial dysfunction, heart dysfunction, apoptosis, cardiomyocytes, ROS

## Abstract

Lipid overload contributes to cardiac complications of diabetes and obesity. However, the underlying mechanisms remain obscure. This study investigates the role of gamma-aminobutyrate transaminase (ABAT), the key enzyme involved in the catabolism of γ-aminobutyric acid (GABA), in lipid overload-induced cardiac injury. Microarray revealed a down-regulation of ABAT mRNA expression in high fat diet (HFD)-fed mouse hearts, which correlated with a reduction in ABAT protein level and its GABA catabolic activity. Transgenic mice with cardiomyocyte-specific ABAT over-expression (Tg-ABAT/tTA) were generated to determine the role of ABAT in lipid overload-induced cardiac injury. Feeding with a HFD to control mice for 4 months reduced ATP production and the mitochondrial DNA copy number, and induced myocardial oxidative stress, hypertrophy, fibrosis and dysfunction. Such pathological effects of HFD were mitigated by ABAT over-expression in Tg-ABAT/tTA mice. In cultured cardiomyocytes, palmitate increased mitochondrial ROS production, depleted ATP production and promoted apoptosis, all of which were attenuated by ABAT over-expression. With the inhibition of ABAT’s GABA catabolic activity, the protective effects of ABAT remained unchanged in palmitate-induced cardiomyocytes. Thus, ABAT protects the mitochondrial function in defending the heart against lipid overload-induced injury through mechanisms independent of its GABA catabolic activity, and may represent a new therapeutic target for lipid overload-induced cardiac injury.

## 1. Introduction

The rising prevalence of obesity and diabetes has been a major public health problem worldwide [1]. Both obesity and diabetes are leading risk factors for cardiovascular disease [2]. The heart uses fatty acid as a primary energy source, with an estimated 60–80% of ATP generated from fatty acid oxidation and the rest of the ATP generated from glucose utilization [3]. A healthy heart has a rapid fatty acid uptake rate, which is tightly coupled with the mitochondrial utilization of fatty acid. Under the conditions of obesity and diabetes, excessive fatty acid uptake exceeds the mitochondrial fatty acid oxidation capacity, leading to lipid overload or the accumulation of lipid intermediates in cardiomyocytes [4], which may change cellular structure and induce cell death, a condition referred to as cardiac lipotoxicity [5]. Lipotoxicity impairs heart function, eventually leading to pathological cardiac remodeling and heart failure. To date, the underlying mechanisms of lipid overload-associated cardiac injury remain incompletely understood.

Gamma-aminobutyrate transaminase (ABAT), also known as γ-aminobutyric acid transaminase (GABA-T), is the key enzyme involved in the catabolism of principal inhibitory neurotransmitter GABA. ABAT is localized within the mitochondrial matrix and catalyzes the transfer of amino group of GABA to α-ketoglutarate, forming succinic semialdehyde, which is subsequently converted to succinate, a key intermediate in the tricarboxylic acid cycle [6]. Interestingly, a recent study reported that ABAT functions in the mitochondrial nucleoside salvage pathway to facilitate the conversion of dNDPs to dNTPs for mitochondrial DNA (mtDNA) biosynthesis in fibroblasts and photoreceptor cells [7]. This study underscores an important role of ABAT in mitochondrial biogenesis. Given that mitochondrial DNA synthesis and biogenesis are compromised in diseased hearts [8,9,10,11], it is possible that ABAT may play a role in cardiac bio-pathology. However, it has never been investigated whether ABAT is altered in diseased hearts and what its potential role is in the heart.

In this study, we generate transgenic mice with cardiomyocyte-specific ABAT over-expression and investigate the roles and potential mechanisms of ABAT in lipid overload-induced heart damage. We use a high fat diet-fed mouse model and an in vitro palmitate incubation model. We found that ABAT over-expression protects against lipid overload-induced cardiomyocyte death and heart dysfunction. Thus, ABAT may represent a new therapeutic target for lipid overload-induced cardiac injury.

## 2. Materials and Methods

### 2.1. Animal

This research conformed to the Guide for the Care and Use of Laboratory Animals published by the U.S. National Institute of Health (NIH Publication, 8th Edition, 2011). All experimental protocols were approved by the Animal Use Subcommittee of Soochow University, China, and Western University, Canada. Breeding pairs of C57BL/6 male mice were purchased from the Jackson Laboratory. A breeding program was implemented to produce neonates at our animal care facilities. Transgenic mice with a cardiomyocyte-specific over-expression of tetracycline transactivator (Tg-tTA) were kindly provided by Dr. Jeffrey Robbins [12]. A transgenic vector containing full-length human *Abat* cDNA (accession number: NM_020686) under the tetracycline transactivator (tTA)-inducible mouse alpha-myosin heavy chain (α-MHC) promoter was constructed, and transgenic mice with human ABAT expression driven by tTA inducible mouse α-myosin heavy chain (α-MHC) promoter (Tg-ABAT) was generated as described [12]. The Tg-ABAT mice were then crossed with Tg-tTA mice to produce wild-type, Tg-tTA, Tg-ABAT, and Tg-ABAT/tTA mice, which were identified by polymerase chain reaction (PCR) using both tTA and human *Abat* primers. Tg-ABAT/tTA mice expressed human ABAT in cardiomyocytes. All of the adult mice used in this study were littermates of the same generation.

All the animals were housed in a temperature- and humidity-controlled facility at 12 h light and dark cycles with water and food *ad libitum*.

### 2.2. Experimental Protocol

Male Tg-ABAT/tTA mice and their littermate controls, including wild-type, Tg-tTA and Tg-ABAT mice, were fed a high fat diet (60% kcal from fat, HFD) or a standard chow diet (normal diet, ND) starting from age 4 weeks for a total of 4 months. This HFD contained 26.2% protein, 26.3% carbohydrate and 34.9% fat (% by weight) (Research Diets, New Brunswick, NJ, USA).

### 2.3. Intraperitoneal Glucose Tolerance Test

For intraperitoneal glucose tolerance test (GTT), mice were fasted for 12 h before being administered a glucose load (2 g/kg, *i.p.*). Blood samples were collected from the tail vein at 0, 15, 30, 60, 120 min after glucose injection, and blood glucose was measured using a glucose meter (Roche, Indianapolis, IN, USA). the GTT curve was obtained and the area under curve (AUC) was calculated.

### 2.4. Echocardiography

The mice were anesthetized with inhaled isoflurane (1%) and imaged using a 40 MHz linear array transducer attached to a preclinical ultrasound system (Vevo 2100, Fujifilm VisualSonics, Toronto, ON, Canada) with nominal in-plane spatial resolution of 40 μm (axial) × 80 μm (lateral). M-mode and 2-D parasternal short-axis scans (133 frames/second) at the level of the papillary muscles were employed to assess the changes in left ventricular (LV) end-systolic inner diameter, LV end-diastolic inner diameter, LV posterior wall thickness in end-diastole and end-systole, and fractional shortening (FS). To assess the diastolic function, the pulsed wave Doppler measurements of maximal early (E) and late (A) transmittal velocities in diastole were obtained in the apical view with a cursor at mitral valve inflow [13].

### 2.5. Histological Analysis

Heart tissues were routinely collected, fixed, processed and sectioned. The cardiomyocyte cross-sectional area and collagen deposition were determined after Texas-Red^TM^-X conjugated wheat germ agglutinin (WGA, Molecular Probes, Eugene, OR, USA) staining and Sirius red (Shanghai Yuanye Biotechnology, Shanghai, China) staining, respectively, as previously described [13].

### 2.6. Analysis of Differentially Expressed Genes

Microarray was performed to analyze the gene expression in ND- and HFD-fed mouse hearts (three hearts in each group) through the collaboration with Oebiotech Inc. (Shanghai, China). Briefly, the total RNA was extracted and purified from heart tissues using the mirVana™ RNA Isolation Kit (Ambion, Austin, TX, USA) and QIAGEN RNeasy^®^ Mini Kit (Qiagen, Valencia, CA, USA), respectively, following the manufacturer’s instructions. cRNA synthesis, labeling, fragmentation and hybridization were sequentially conducted using the Agilent Mouse Gene Expression Microarray Kit (Design ID: 028005, Agilent Technologies, Santa Clara, CA, USA). The slides were scanned using the Agilent Microarray Scanner (Agilent p/n G2505C, Agilent Technologies, Santa Clara, CA, USA). Data was extracted using the Agilent Feature Extraction Software (Version 10.7, Agilent Technologies, Santa Clara, CA, USA). The mRNA expression profiles were deposited into the gene expression omnibus (GEO) database (GSE150229).

### 2.7. Triglyceride in Heart Tissues

Triglyceride was measured in heart tissue lysates using a commercially available kit (Nanjing Jiancheng Bioengineering Institute, Nanjing, China), according to the manufacturer’s instructions.

### 2.8. Determination of Oxidative Stress in Heart Tissues

The formation of reactive oxygen species (ROS) in heart tissue lysates was assessed using the Amplex^®^ Red Hydrogen Peroxide/Peroxidase Assay Kit (Molecular Probes, Eugene, OR, USA). Oxidative damage was determined by measuring the malondialdehyde (MDA) and protein carbonyl content in heart tissue lysates using a TBARS assay kit (Cayman Chemical, Ann Arbor, MI, USA) and a Protein Carbonyl Colorimetric Assay Kit (Cayman Chemical, Ann Arbor, MI, USA), respectively. All these experiments were conducted following the manufacturer’s instructions.

### 2.9. Isolation and Cultures of Neonatal Cardiomyocytes

Neonatal mice (born within 24 h) were euthanized by decapitation and their hearts were excised. Neonatal cardiomyocytes were isolated and cultured according to the methods we described previously [5]. After 24 h of isolation, the cardiomyocytes were subjected to various treatments.

### 2.10. Bovine Serum Albumin (BSA)-Free Fatty Acid (FFA) Conjugation

Sodium oleate and sodium palmitate (Sigma-Aldrich, St. Louis, MO, USA) were dissolved in purified water at 70 °C to a concentration of 20 mM, and then conjugated with 20% BSA (Sigma-Aldrich, St. Louis, MO, USA) with a volume ratio of 1:1 at 37 °C. For cell treatment, BSA-FFA conjugates were diluted to the concentration of 500 μM with cell culture medium.

### 2.11. Adenoviral Infection of Cardiomyocytes

For in vitro ABAT over-expression, neonatal cardiomyocytes were infected with an adenoviral vector containing human *Abat* (Ad-ABAT, Vector Biolabs, Philadelphia, PA, USA) at a multiplicity of infection of 100 PFU/cell. An adenoviral vector containing beta-gal (Ad-gal, Vector Biolabs, Philadelphia, PA, USA) served as a control. Adenovirus-mediated gene transfer was implemented as previously described [14].

### 2.12. Measurement of Mitochondrial Superoxide Generation in Cardiomyocytes

Mitochondrial superoxide generation was assessed in living cardiomyocytes using the MitoSOX^TM^ Red mitochondrial superoxide indicator (Molecular Probes, Eugene, OR, USA) following the manufacturer’s instructions.

### 2.13. ABAT’s GABA Catabolic Activity

ABAT activity assay was performed as previously described [15], with minor modification. Briefly, tissues or cells were homogenized with a lysis buffer (10 mM K_2_HPO_4_, 20% glycerol, 0.13% Triton X-100, 0.1 mM glutathione, 0.1 mM pyridoxal-5′-phosphate, and 1 mM disodium EDTA). After centrifugation at 12,000× *g* at 4 °C for 30 min, the supernatant containing 80 μg of total protein, or lysis buffer alone as a blank control, were mixed with 180 μL of assay buffer (100 mM potassium pyrophosphate, 5 mM α-ketoglutarate, 0.4 mM NAD^+^, 2 mM DTT, and 0.01 mM PLP). After pre-incubation at 30 °C for 15 min, GABA was added to the mixtures to a final concentration of 10 mM and incubated for 30 min. ABAT activity was proportional to the formation of NADH from NAD^+^, which was recorded as the increase in absorbance at 340 nm.

### 2.14. Analysis of Apoptosis

Apoptosis was determined by measuring caspase 3 activity and cellular DNA fragmentation as previously described [16].

### 2.15. Real-Time RT-PCR

Total RNA was extracted using the TRIzol reagent (Takara, Tokyo, Japan) following the manufacturer’s instructions. Real-time reverse transcription (RT)-PCR was performed as previously described [17]. The sequences of primers are as follows: *Abat*, 5′-GGAGCATCGGAAGGTGATCG-3′ and 5′-CCCGCGTCCTGATTAGATGG-3′; *Gapdh*, 5′-CAGTGTTGGGGGCTGAGTTC-3′ and 5′-AAAGGGCATCCTGGGCTACA-3′.

For the determination of mitochondrial DNA (mtDNA) copy number, total DNA was isolated from heart tissues using QIAamp DNA Mini Kit (Qiagen, Valencia, CA, USA) following the manufacturer’s instructions. Mitochondrially encoded NADH1 (*mtND1*) and beta-2-microglobulin (*B2M*) DNA was measured by real-time PCR and their ratio was used as an indicator of the mtDNA copy number. The sequences of the primers for *mtND1* and *B2M* are as follows: *mtND1*, 5′-GAGGGAACCAAACTGAACGC-3′ and 5′-TGGATCCGTTCGTAGTTGGAG-3′; and *B2M*, 5′-CAGACTCTGCGATGTTTCCA-3′ and 5′-GCCTGAGCACTTCCAGAAAC-3′.

### 2.16. Western Blotting

Tissue and cell lysates were subjected to SDS-PAGE for separation of proteins followed by immuno-blotting as previously described [18]. Anti-ABAT (1:10,000 dilution; ab108249, Abcam, Cambridge, MA, USA) and anti-GAPDH (1:5000 dilution; 10494-1-AP, Proteintech, Chicago, IL, USA) were used to detect the protein levels of ABAT and GAPDH, respectively.

### 2.17. Statistical Analysis

Data were expressed as the mean ± SD. Student’s *t*-test was employed for comparison between the two groups. ANOVA followed by the Newman–Keuls test was performed for multi-group comparisons. A *p*-value of less than 0.05 was considered significant.

## 3. Results

### 3.1. HFD Feeding Results in a Down-Regulation of ABAT in Mouse Hearts

**Figure 1:**
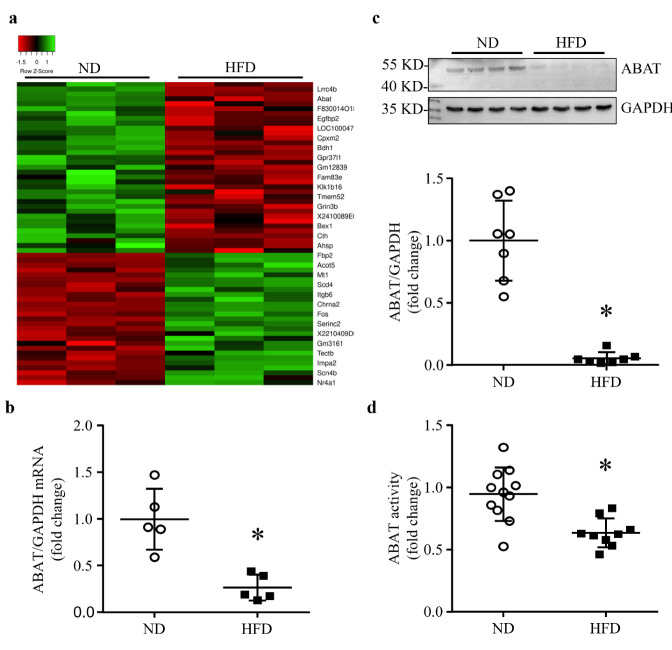
HFD results in a reduction of ABAT in mouse hearts. After 4 months of high fat diet (HFD) feeding, cDNA microarray determined differential expression of genes in mouse hearts. (**a**) Heat map of differential gene expression. (**b**) The mRNA level of *Abat* relative to GAPDH was analyzed by RT-PCR. Data are mean ± SD, n = 5 per group. * *p* < 0.05 vs. ND (normal diet). (**c**) ABAT protein level was detected by western blot analysis. Upper panel: a representative western blot for ABAT and GAPDH from 4 out of 7 different hearts in each group. Bottom panel: quantification of ABAT/GAPDH ratio. Data are mean ± SD, n = 7 per group. * *p* < 0.05 vs. ND. (**d**) ABAT activity was measured by an enzymatic activity assay. Data are mean ± SD, n = 9–11 per group. * *p* < 0.05 vs. ND.

Microarray analysis identified the difference of gene expression between ND- and HFD-fed mouse hearts (Figure 1a). Among the differentially expressed genes, *Abat* was one of the most down-regulated genes in the HFD group compared with the ND group (Figure 1a). The down-regulation of *Abat* mRNA was validated by real-time RT-PCR (Figure 1b). Similarly, the protein levels of ABAT and its GABA catabolic activities were significantly lower in the HFD-fed group compared with the ND-fed mouse heart tissues (Figure 1c,d). Since HFD increased the triglyceride contents in mouse heart tissues (Supplementary Figure S1a), indicative of myocardial ectopic lipid accumulation, these results indicate that lipid overload reduced ABAT in the hearts of HFD-fed mice.

### 3.2. Cardiomyocyte-Specific Over-Expression of ABAT Reduces Myocardial Dysfunction without Affecting the Systemic Metabolism in Mice Fed a HFD

**Figure 2:**
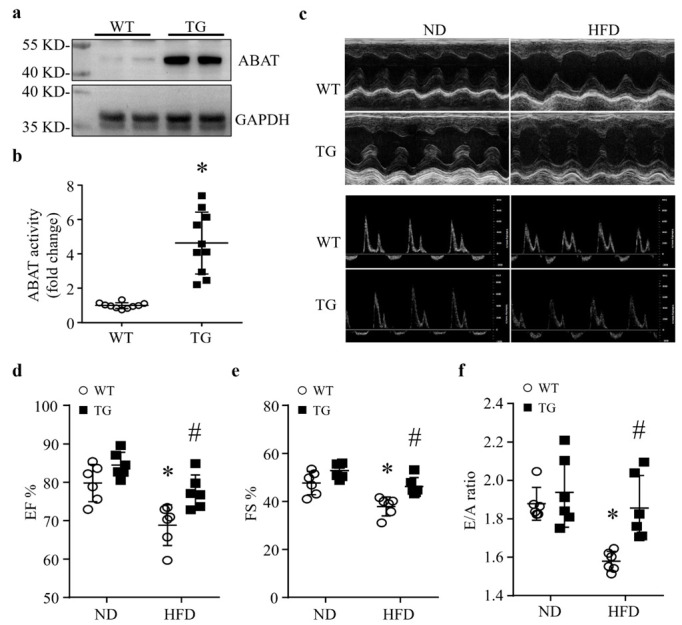
Cardiac overexpression of ABAT protects myocardial function in HFD fed mice. Transgenic mice with ABAT over-expression (TG) and their littermate controls (WT) were fed a ND (normal diet) or HFD (high fat diet) for 4 months. (**a**) A representative western blot for ABAT and GAPDH from 2 different hearts in each group. (**b**) ABAT’s GABA catabolic activity in the heart. Data are mean ± SD, n = 10 per group. * *p* < 0.05 vs. WT. (**c**–**f**) Myocardial function was measured by echocardiography. (**c**) Representative images at the papillary muscle level of echocardiography. (**d**) Ejection fraction (EF%). (**e**) Fractional shortening (FS%). (**f**) E/A ratio. Data are mean ± SD, n = 6 per group. * *p* < 0.05 vs. ND + WT, # *p* < 0.05 vs. HFD + WT.

A reduction in ABAT in HFD-fed mouse hearts suggests a potential role of ABAT in lipid overload-induced myocardial disorders. To address this, we created a novel line of transgenic mice with cardiac-specific over-expression of ABAT (Tg-ABAT/tTA, Supplementary Figure S2). The over-expression of ABAT was verified in the heart tissues of Tg-ABAT/tTA mice (aged 5 months) by Western blot analysis (Figure 2a). The GABA catabolic activity assay confirmed an up-regulation of ABAT activity by about 5-fold in Tg-ABAT/tTA mouse hearts (Figure 2b). In contrast, ABAT protein expression was not increased in other organs of Tg-ABAT/tTA mice compared with their littermate controls, including lung, liver and kidney tissues (Supplementary Figure S3).

Under normal conditions, transgenic Tg-ABAT/tTA mice grew normally without adverse phenotypes and there are no differences in body weight, metabolism and myocardial function between Tg-ABAT/tTA mice and their relevant littermate controls by the age of 5 months (Figure 2c–f and Supplementary Figure S1a–d). These results rule out the potential adverse effects of ABAT over-expression and tTA transgene expression in Tg-ABAT/tTA mice.

To determine the effects of transgenic ABAT over-expression in lipid overload-associated myocardial disorders, we fed Tg-ABAT/tTA mice and their littermate controls a HFD or ND starting from the age of 1 month for a total of 4 months, as previously described [5]. Mice fed a HFD developed metabolic disorders, including increased triglyceride levels, increased body weight, elevated fasting blood glucose and impaired glucose tolerance when compared to ND-fed mice (Supplementary Figure S1a–d and Supplementary Table S1). Transgenic ABAT over-expression did not change the metabolic parameters in mice fed an ND or HFD. No death was observed in mice fed an ND or HFD for 4 months.

The echocardiographic analysis revealed that mice fed a HFD displayed a decrease in EF% and FS%, indicative of an impaired systolic function, and a decrease in the E/A ratio, indicative of a compromised diastolic function, suggesting that lipid overload induces myocardial dysfunction in control mice (Figure 2c–f and Supplementary Table S2), which is consistent with previous reports [5]. However, transgenic ABAT over-expression relatively increased the EF%, FS% and E/A ratio in Tg-ABAT/tTA mice compared with their littermate controls after HFD feeding. Since our pilot study showed that myocardial function was similar among wild-type, Tg-tTA and Tg-ABAT fed an ND and HFD, we used Tg-ABAT mice as their littermate controls for Tg-ABAT/tTA mice for the following studies. Thus, our results suggest that transgenic ABAT over-expression in hearts reduces lipid overload-induced myocardial dysfunction in mice.

### 3.3. Lipid Overload-Induced Myocardial Remodeling Is Attenuated by Transgenic ABAT Over-Expression in Mice

**Figure 3:**
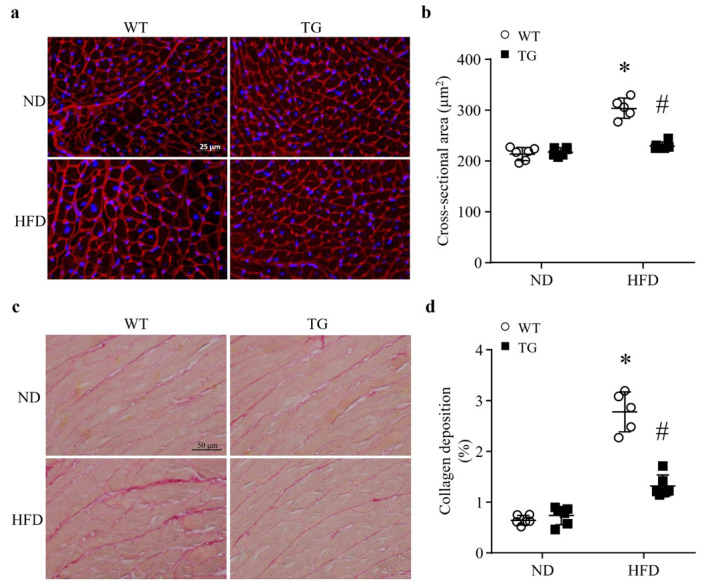
Cardiomyocyte cross-sectional area and collagen deposition in the heart. Transgenic mice with ABAT over-expression (TG) and their littermate controls (WT) were fed a ND (normal diet) or HFD (high fat diet) for 4 months. (**a**) Representative microphotographs of Texas Red^TM^-X conjugated wheat germ agglutinin (WGA) staining (red) and nucleus staining with Hoechst33324 (blue). (**b**) Quantification of cardiomyocyte cross-sectional area. Data are mean± SD. n = 5–6 per group. * *p* < 0.05 vs. ND + WT, # *p* < 0.05 vs. HFD + WT. (**c**) Representative microphotographs of Sirius red staining. (**d**) Quantification of collagen deposition. Data are mean± SD. n = 5–6 per group. * *p* < 0.05 vs. ND + WT, # *p* < 0.05 vs. HFD + WT.

Four months after feeding a HFD, the histological analysis of cardiomyocyte cross-sectional areas revealed an increase in cardiomyocyte size, indicative of cardiomyocyte hypertrophy in control mice. However, the cardiomyocyte sizes were not increased in Tg-ABAT/tTA mice fed a HFD (Figure 3a,b). There was no difference in the cardiomyocyte size when comparing Tg-ABAT/tTA mice and their littermate controls after feeding an ND. These results demonstrate that transgenic ABAT over-expression reduces HFD-induced myocardial hypertrophy.

To determine myocardial fibrosis, we stained heart tissue sections with Picro Sirius red to highlight collagen deposition. The deposition of total collagen was increased in HFD-fed mouse hearts as determined by the ratio of collagen area to total area; however, transgenic ABAT over-expression correlated with a significant reduction in the levels of collagen deposition in Tg-ABAT/tTA mice fed a HFD (Figure 3c,d). This result suggests that ABAT prevents myocardial fibrosis in mice fed a HFD.

### 3.4. Transgenic ABAT Over-Expression Improves Mitochondrial Function and Attenuates Oxidative Stress and Apoptosis in HFD-Fed Mouse Hearts

**Figure 4:**
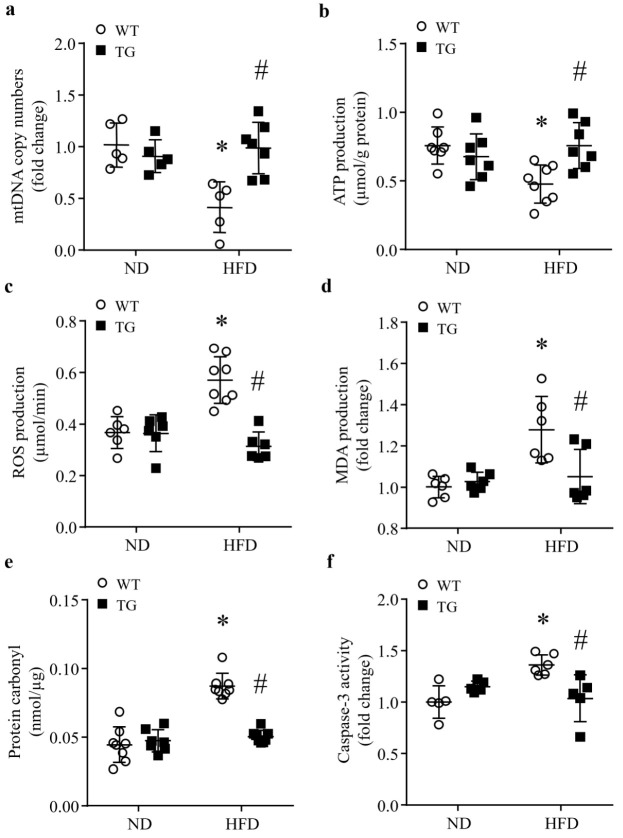
Effects of transgenic ABAT over-expression on mitochondrial function, oxidative stress and caspase-3 activation in HFD-fed mouse hearts. Transgenic mice with ABAT over-expression (TG) and their littermate controls (WT) were fed a ND (normal diet) or HFD (high fat diet) for 4 months. (**a**) Mitochondrial DNA (mtDNA) copy numbers in the heart. Data are mean± SD. n = 5–7 per group. * *p* < 0.05 vs. ND + WT, # *p* < 0.05 vs. HFD + WT. (**b**) ATP production in the heart. Data are mean± SD. n = 7–8 per group. * *p* < 0.05 vs. ND + WT, # *p* < 0.05 vs. HFD + WT. (**c**–**e**) Assessment of oxidative stress in the heart. Data are mean ± SD. n = 6–8 per group. * *p* < 0.05 vs. ND + WT, # *p* < 0.05 vs. HFD + WT. (**c**) ROS production, (**d**) MDA production and (**e**) protein carbonyl. (**f**) Apoptosis was assessed by caspase-3 activity. Data are mean± SD. n = 5–6 per group. * *p* < 0.05 vs. ND + WT, # *p* < 0.05 vs. HFD + WT.

Since ABAT was reported to play an important role in maintaining mtDNA synthesis [7], we analyzed the mtDNA copy number and cellular energy production in the form of ATP in heart tissues. As shown in Figure 4a,b, mtDNA copy number and ATP production were significantly reduced in control mice fed a HFD relative to ND. However, their levels were preserved in Tg-ABAT/tTA mice fed a HFD. ATP production and mtDNA copy number were similar in Tg-ABAT/tTA mice and their littermate controls fed an ND. This result suggests that the over-expression of ABAT may protect mitochondrial function in lipid overload-induced mice.

Compromised mitochondria ensure excessive ROS production, leading to oxidative stress. Therefore, we determined whether transgenic ABAT over-expression could ameliorate HFD-induced oxidative stress in the heart. As shown in Figure 4c–e, HFD induced a significant increase in ROS production in control mouse heart tissues, which was concomitant with myocardial oxidative damage as evidenced by the increased production of MDA and protein carbonyls. The productions of ROS, MDA and protein carbonyls were much less in heart tissues of Tg-ABAT/tTA mice, compared with their littermate controls after feeding a HFD. ND-fed Tg-ABAT/tTA mice and their littermate controls had similar productions of ROS, MDA and protein carbonyls in their heart tissues. Thus, transgenic ABAT over-expression prevents oxidative stress in HFD-fed mouse hearts.

It is well known that oxidative stress promotes apoptotic cell death [19], an important mechanism contributing to lipid overload-associated heart dysfunction, we determined caspase-3 activity as an indicator of apoptosis in the heart. Caspase-3 activity was much higher in mice fed a HFD relative to ND. However, transgenic ABAT over-expression significantly reduced HFD-induced caspase-3 activity in Tg-ABAT/tTA mice compared with their littermate controls (Figure 4f). This result suggests that the over-expression of ABAT may prevent lipid overload-induced apoptotic cell death in the heart.

### 3.5. Over-Expression of ABAT Attenuates Palmitate-Induced Cardiomyocyte Injury In Vitro

**Figure 5:**
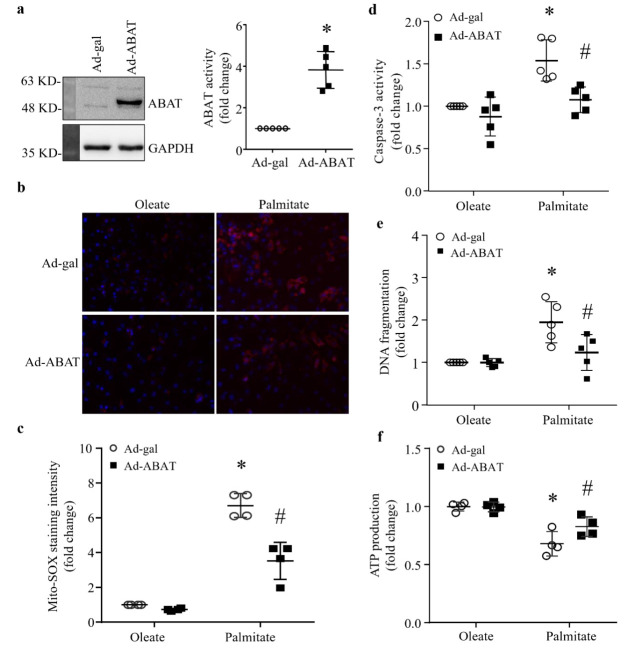
Effects of ABAT over-expression in palmitate-incubated neonatal mouse cardiomyocytes. Cultured neonatal mouse cardiomyocytes were infected with an adenoviral vector expressing ABAT (Ad-ABAT) or beta-gal (Ad-gal) and then exposed to palmitate or oleate (0.5 mM for 48 h). (**a**) ABAT protein expression and activity in cardiomyocytes. Left panel: a representative western blot for ABAT and GAPDH. Right panel: ABAT activity. Data are mean ± SD. n = 5 per group. * *p* < 0.05 vs. Ad-gal. (**b**) Representative microphotographs of MitoSOX^TM^ Red staining (red) and nucleus staining with Hoechst33324 (blue). (**c**) Quantification of MitoSOX^TM^ Red staining intensity. Data are mean ± SD. n = 4 per group. * *p* < 0.05 vs. Oleate + Ad-gal. # *p* < 0.05 vs. Palmitate + Ad-gal. (**d**) Caspas-3 activity. Data are mean ± SD. n = 5 per group. * *p* < 0.05 vs. Oleate + Ad-gal. # *p* < 0.05 vs. Palmitate + Ad-gal. (**e**) DNA fragmentation. n = 5 per group. * *p* < 0.05 vs. Oleate + Ad-gal. # *p* < 0.05 vs. Palmitate + Ad-gal. (**f**) ATP production. n = 4 per group. * *p* < 0.05 vs. Oleate + Ad-gal. # *p* < 0.05 vs. Palmitate + Ad-gal.

To provide direct evidence to support the protective role of ABAT and exclude any confounding effects of systemic factors in attenuating lipid overload-induced heart dysfunction, we infected cultured neonatal mouse cardiomyocytes with Ad-ABAT. Ad-gal served as a control. Twenty-four hours after adenoviral infection, cardiomyocytes were incubated with palmitate or oleate (500 μM) as an osmotic control for another 48 h. We chose palmitate to induce lipid overload in cultured cardiomyocytes, because palmitate is one of the major saturated fatty acids in the plasma [20] and HFD elevates palmitate levels in mouse hearts [2]. Infection with Ad-ABAT increased the levels of ABAT protein and activity in cardiomyocytes (Figure 5a). Incubation with palmitate increased mitochondrial ROS production determined by mito-SOX assay (Figure 5b,c) and induces apoptosis, as evidenced by the increases in caspase-3 activity and cellular DNA fragmentation relative to oleate (Figure 5d,e), all of which were attenuated by ABAT over-expression. The over-expression of ABAT also increased ATP production in palmitate-induced cardiomyocytes (Figure 5f). These results demonstrate that the over-expression of ABAT improves mitochondrial energy metabolism, attenuates mitochondrial ROS production and apoptotic cell death in cardiomyocytes induced by lipid overload.

### 3.6. The Protective Effect of ABAT Over-Expression on Palmitate-Induced Apoptosis Is Independent of Its GABA Catabolic Activity

**Figure 6:**
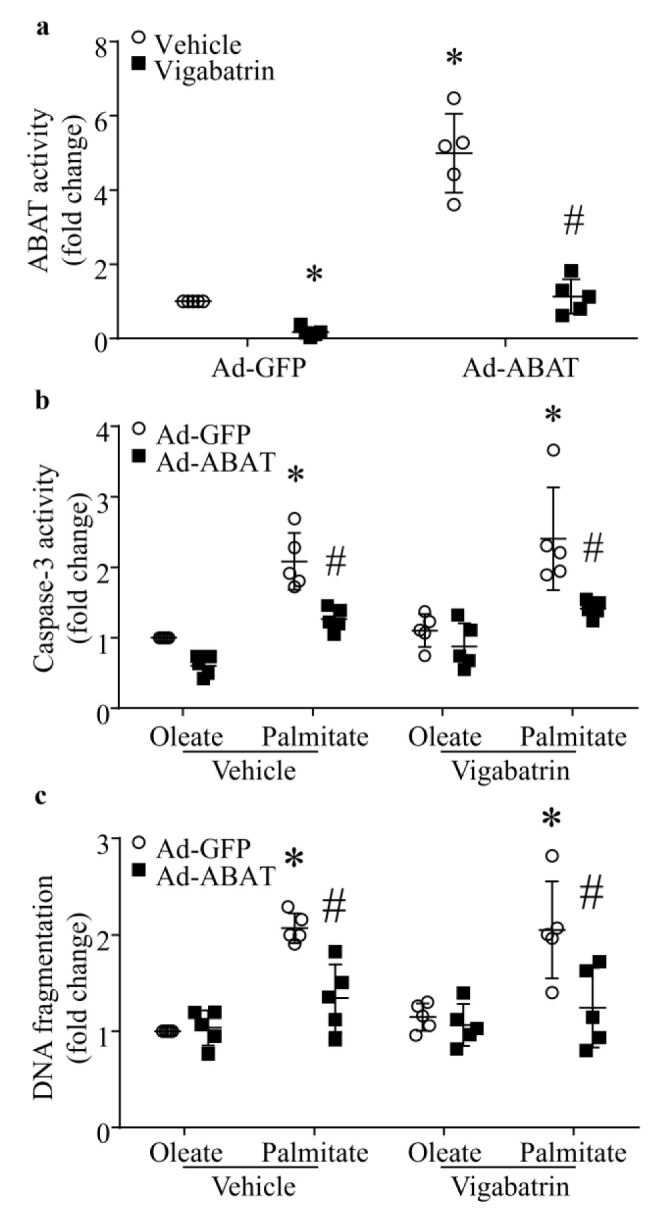
Effects of Vigabatrin on apoptosis in cardiomyocytes. Cultured neonatal mouse cardiomyocytes were infected with an adenoviral vector expressing ABAT (Ad-ABAT) or GFP (Ad-GFP) and then exposed to palmitate or oleate (0.5 mM) in the presence of vehicle or Vigabatrin (500 μM), a selective inhibitor of ABAT for 48 h. (**a**) ABAT’s GABA catabolic activity. Data are mean ± SD, n = 5 in each group. * *p* < 0.05 vs. Ad-GFP + Vehicle, # *p* < 0.05 vs. Ad-ABAT + Vehicle. (**b**) Caspase-3 activity. Data are mean ± SD, n = 5 in each group. * *p* < 0.05 vs. Ad-GFP + Oleate + Vehicle, or Ad-GFP + Oleate + Vigabatrin, # *p* < 0.05 vs. Ad-GFP + Palmitate + Vehicle, or Ad-GFP + Palmitate + Vigabatrin. (**c**) DNA fragmentation. Data are mean ± SD, n = 5 in each group. * *p* < 0.05 vs. Ad-GFP + Oleate + Vehicle, or Ad-GFP + Oleate + Vigabatrin, # *p* < 0.05 vs. Ad-GFP + Palmitate + Vehicle, or Ad-GFP + Palmitate + Vigabatrin.

To gain insights into the role of ABAT, we determined if ABAT’s GABA catabolic activity is involved in lipid overload-induced cardiomyocyte apoptosis and myocardial dysfunction. Neonatal mouse cardiomyocytes were infected with Ad-ABAT or Ad-gal, and 24 h later, incubated with palmitate or oleate (500 µM) in the presence of Vigabatrin (500 µM), a selective inhibitor of ABAT’s GABA catabolic activity [21], or vehicle for additional 48 h. As shown in Figure 6a, ABAT activity was significantly increased in Ad-ABAT compared with Ad-gal-infected cardiomyocytes. Incubation with Vigabatrin resulted in a dramatic reduction of ABAT activity in both Ad-ABAT- and Ad-gal-infected cardiomyocytes. Similarly, palmitate elicited apoptosis as determined by caspase-3 activity and DNA fragmentation in Ad-gal-infected cardiomyocytes. Infection with Ad-ABAT attenuated palmitate-induced apoptosis. Intriguingly, the inhibition of ABAT’s GABA catabolic activity with Vigabatrin had no effect on apoptosis in both palmitate- and oleate-stimulated cardiomyocytes. Furthermore, the protective effect of ABAT over-expression was not changed in palmitate-induced cardiomyocytes when ABAT’s GABA catabolic activity was inhibited with Vigabatrin (Figure 6b,c). This finding suggests that the protective role of ABAT is mediated through mechanisms independent of ABAT’s GABA catabolic activity in lipid overload-induced myocardial injury.

## 4. Discussion

The defect of ABAT is associated with severe neurological disorders, such as early onset encephalopathy and epilepsy [22]. Additionally, GABAergic signaling has been reported to present in non-neuronal tissues. For example, ABAT is down-regulated in clear cell renal carcinoma tissues [23] and basal-like breast cancer tissues [6], and over-expression of ABAT suppresses tumor cell growth [23]. Despite the presence of GABA and GABA receptor in the heart [24], the role of ABAT has never been reported in cardiac bio-pathology. In the current study, we found that lipid overload resulted in a reduction in ABAT in the heart and over-expression of ABAT attenuated lipid overload-induced cardiomyocyte death and heart dysfunction. To the best of our knowledge, this is the first study that reveals the importance of ABAT in defending the heart against lipid overload-induced cardiac pathology.

ABAT is the rate-limiting enzyme that converts GABA to succinate, which subsequently enters the tricarboxylic acid cycle to facilitate mitochondrial oxidative phosphorylation [25]. In fact, it was reported that the disruption of the GABA catabolism impaired mitochondrial respiration in fungus [26] and that the over-expression of ABAT increased, whereas the inhibition of ABAT reduced mitochondrial respiration in articular chondrocytes [27]. As we showed that ABAT was reduced in mouse hearts in response to lipid overload, we subsequently generated a new line of transgenic mice with cardiomyocyte-specific ABAT over-expression to determine the role of ABAT in lipid overload-induced heart dysfunction. We provided in vivo evidence that the over-expression of ABAT increased ATP production reduced oxidative stress, prevented apoptosis, attenuated myocardial remodeling and preserved heart function in HFD-fed mice. In an in vitro model, ABAT over-expression attenuated mitochondrial ROS production and apoptosis, and increased ATP production in palmitate-induced cultured cardiomyocytes. However, the inhibition of ABAT with Vigabatrin did not affect the protective effect of ABAT over-expression on palmitate-induced apoptosis in cardiomyocytes. Thus, we argue that ABAT protects against lipid overload-induced cardiomyocyte death and heart dysfunction through unknown mechanisms independent of its GABA catabolic function. Our data also suggest that GABA catabolism may be a neglectable source of succinate in cardiomyocytes as major sources of succinate for the tricarboxylic acid cycle are available including glucose and lipid metabolism. Nevertheless, the underlying mechanisms by which ABAT exerts cardiac protection require further investigation.

A recent study reveals a new role of ABAT in the mitochondrial DNA salvage pathway, which is independent of the GABAergic system [28]. We determined the effect of ABAT over-expression on the mtDNA copy number in the heart. We showed that lipid overload dramatically decreased the cardiac mtDNA copy number, and that cardiac specific ABAT over-expression prevented mtDNA depletion in HFD-fed mouse hearts. This finding suggests a role of ABAT in maintaining cardiac mitochondrial genome stability in the context of lipid overload-induced heart dysfunction. This raises such a possibility that maintaining the mtDNA copy number through the mtDNA salvage pathway and subsequently mitochondrial biogenesis, which is compromised in the heart under metabolic disorders [29], may be a mechanism by which ABAT protects the heart under lipid overload. However, our data does not support this hypothesis, as the inhibition of ABAT’s GABA catabolic activity did not reverse the protective effect of ABAT over-expression on palmitate-induced apoptosis in cardiomyocytes, whereas ABAT requires its GABA catabolic function for mitochondrial nucleoside metabolism [7]. Thus, it seems that the role of ABAT in the mitochondrial DNA salvage pathway may depend on different cells/tissues and/or pathological conditions.

In mitochondria, there is the interplay among mtDNA deficiency, respiration and oxidative stress. Since mtDNA is responsible for coding of several subunits of respiratory complex, deficiency of mtDNA results in aberrant oxidative phosphorylation and excessive ROS production due to an imbalance between subunits of the electron transport chains coded by mtDNA and those coded by nuclear DNA [30]. In turn, excessive ROS reacts with the sugar phosphate backbone and pyrimidine bases, causing mtDNA breaks and base modifications [31], which further impairs mitochondrial function, leading to further ROS production. Likewise, excessive ROS impairs respiration in the mitochondria. It is speculated that ABAT may prevent mitochondrial ROS production or protect respiration thereby reducing lipid overload-induced cardiomyocyte death and heart dysfunction.

## 5. Conclusions

We demonstrated a new role of ABAT in protecting against lipid overload-induced cardiomyocyte death and heart dysfunction, which is associated with preservation of mitochondrial function and energy metabolism, and prevention of oxidative stress. Thus, ABAT may represent a new therapeutic target for metabolic disorder-related heart diseases. Future studies are needed to determine whether ABAT plays similar roles in other heart diseases. It is important to point out that the beneficial role of ABAT is independent of its GABA catabolic function. An understanding of the underlying mechanisms may provide important insights into ABAT biology and its implications in patho-physiology.

## Data Availability

The microarray data are available in GEO database (GSE150229). Other data presented in this study are available on request from the authors.

## Supplementary Materials

The following supporting information can be downloaded at https://www.mdpi.com/article/10.3390/ijms23042182/s1.
