# Supplementary material for: Gamma-Aminobutyrate Transaminase Protects against Lipid Overload-Triggered Cardiac Injury in Mice
Source: Int J Mol Sci. 2022 Feb 16;23(4):2182. doi: 10.3390/ijms23042182 (PMC8874535; doi:10.3390/ijms23042182)
Supplement: Supplementary file 1 [file ijms-23-02182-s001.zip › ijms-1582490-supplementary.pdf]

International Journal of
Molecular Sciences

MDPI

Article

Gamma-Aminobutyrate Transaminase Protects Against Lipid Overload-Triggered Cardiac Injury in Mice

Mengxiao Zhang [1,2,3,†], Huiting Zhong [1,†], Ting Cao [1], Yifan Huang [1], Xiaoyun Ji [3,4], Guo-Chang Fan [5] and Tianqing Peng [3,4,6,7,*]

[1] Institutes of Biology and Medical Sciences, Soochow University, Suzhou 215123, China; zmx1088@163.com (M.Z.); zht0905zht@163.com (H.Z.); tcao704@163.com (T.C.); yyzhyf@hotmail.com (Y.H.)
[2] School of Pharmacy, Bengbu Medical College, Bengbu 233000, China
[3] Department of Pathology and Laboratory Medicine, Western University, London, ON N6A 5C1, Canada; xji86@uwo.ca
[4] Lawson Health Research Institute, London Health Sciences Centre, London, ON N6A 5W9, Canada
[5] Department of Pharmacology and Systems Physiology, University of Cincinnati College of Medicine, Cincinnati, OH 45267, USA; fangg@ucmail.uc.edu
[6] Department of Medicine, Western University, London, ON N6A 5W9, Canada
[7] VRLA6 -140, 800 Commissioners Road, London, ON N6A 5W9, Canada
* Correspondence: tpeng2@uwo.ca; Tel.: +1-519-6858500-55441
† These authors equally contributed to this work.

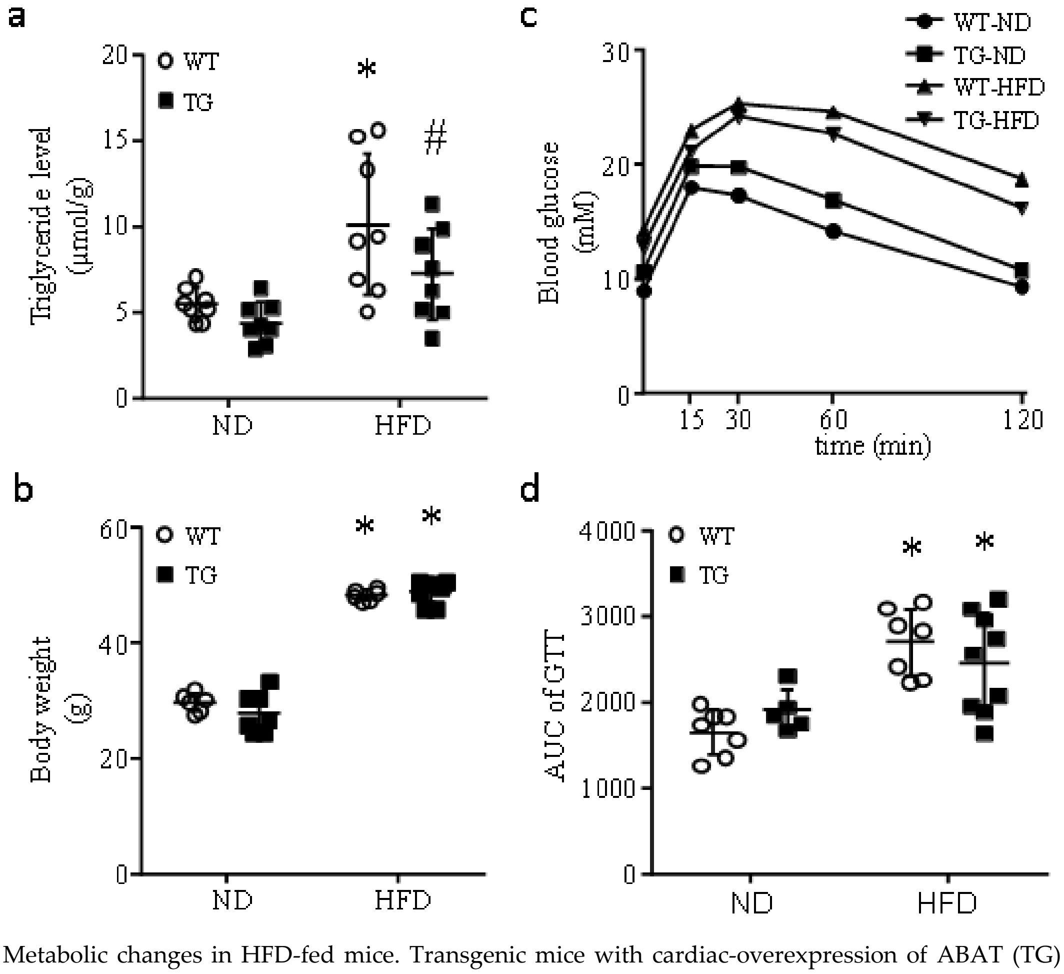

Figure S1. Metabolic changes in HFD-fed mice. Transgenic mice with cardiac-overexpression of ABAT (TG) and their littermate controls were fed with a HFD or ND for 4 months. (a) Triglyceride levels in heart tissues. (b) Body weight. (c) Glucose tolerance test (GTT) curve. (d) Area under curve (AUC) of GTT. Data are mean±SD, n=5-9 in each group. $^{*}P<0.05$ vs. ND+WT or ND+TG, $^{\#}P<0.05$ vs. HFD+WT.

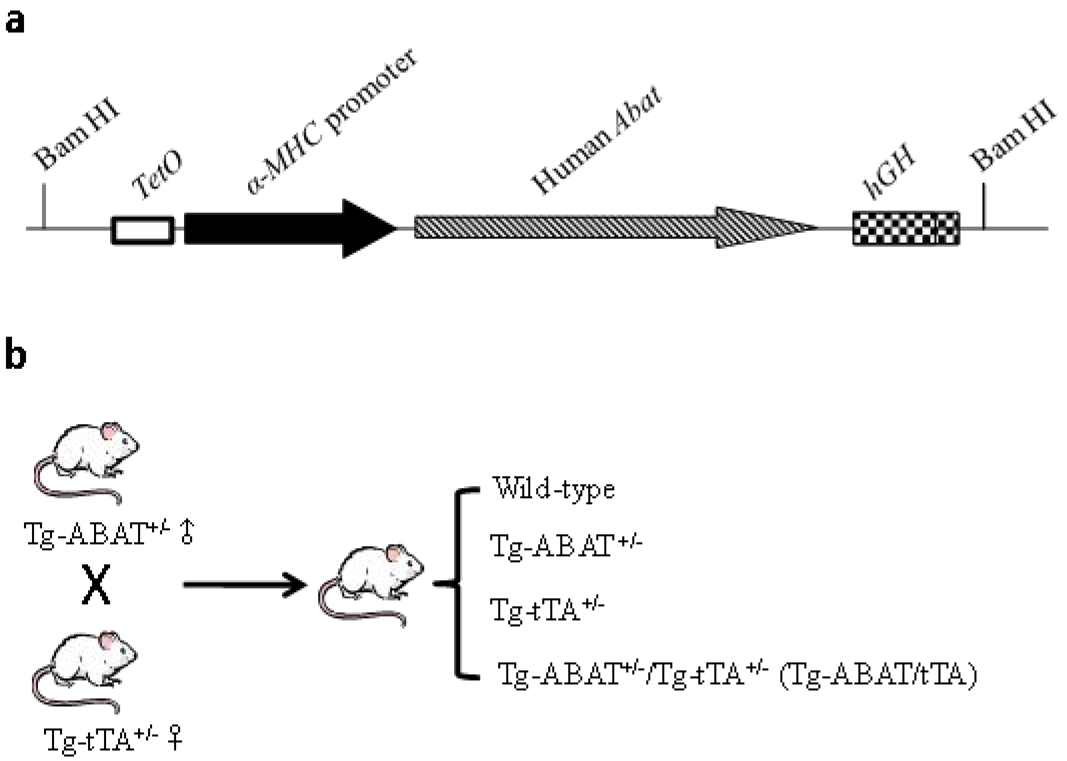

Figure S2. Generation of transgenic mice with cardiomyocyte-specific over-expression of human ABAT. (a) Schematic structure of the transgenic vector containing full-length human Abat cDNA (coding region) under the tetracycline transactivator (tTA)-inducible mouse alpha-myosin heavy chain (α-MHC) promoter. (b) The Tg-ABAT$^{+/-}$ mice were crossed with transgenic mice with cardiomyocyte-specific tTA overexpression (Tg-tTA$^{+/-}$) to produce wild-type, Tg-tTA$^{+/-}$, Tg-ABAT$^{+/-}$ and Tg-ABAT$^{+/-}$/tTA$^{+/-}$ mice. The double Tg-ABAT$^{+/-}$/tTA$^{+/-}$ mice expressed human ABAT restricted to cardiomyocytes and were used as transgenic mice.

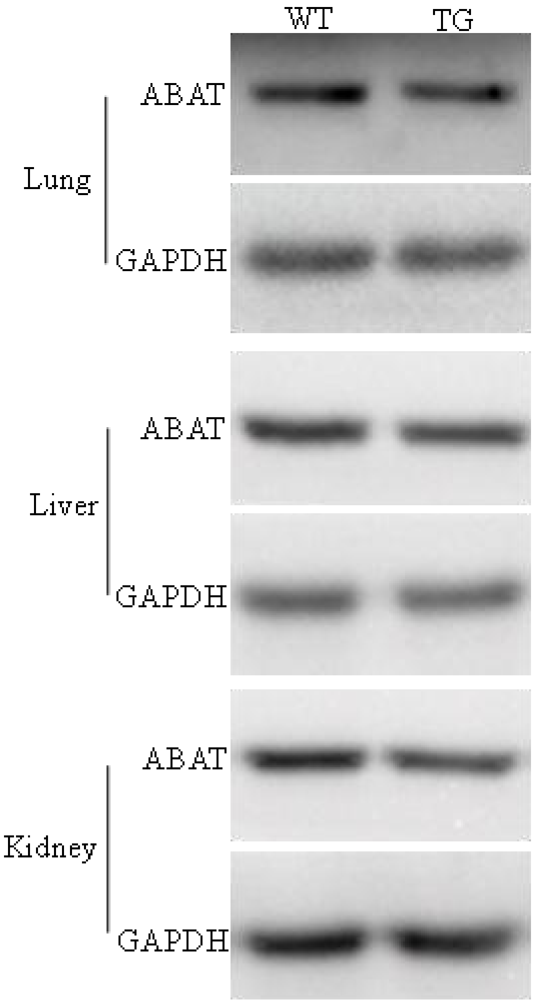

Figure S3. ABAT protein expression in lung, liver and kidney tissues. Lung, liver and kidney tissues from transgenic mice with cardiomyocyte-specific ABAT over-expression (TG) and their littermate controls (WT) were collected and subjected to western blot analysis for ABAT and GAPDH protein. Representative western blots for ABAT and GAPDH show that the protein levels of ABAT do not differ between transgenic mice and their littermate controls.

Table S1. Fasting blood glucose of mice fed a HFD for 4 months

Group	n	Fasting blood glucose (mM)
Control-ND	7	9.00±1.02
Tg-ABAT/tTA-ND	6	10.57±2.70
Control-HFD	7	14.21±2.80*
Tg-ABAT/tTA-HFD	9	12.81±2.22

ND, normal diet; HFD, high fat diet. Data are mean ± SD, n=6-9, *$P<0.05$ vs. Control-ND.

Table S2. Echocardiographic analysis in mice fed a high fat diet for 4 months

	Control-ND	Tg-ABAT/tTA-ND	Control-HFD	Tg-ABAT/tTA-HFD
E/A	1.88±0.08	1.94±0.18	1.58±0.05*	1.86±0.17#
FS (%)	47.66±4.89	52.84±3.17	37.96±3.90*	46.25±3.77#†
EF (%)	79.79±4.84	84.50±3.32	68.83±5.27*	77.50±4.41#†
$LVID_d$ (mm)	3.64±0.21	3.52±0.21	4.01±0.33	3.83±0.37
$LVID_s$ (mm)	1.97±0.20	1.89±0.26	2.37±0.28	2.01±0.39

E/A, the ratio of E over A; FS, fractional shortening; EF, ejection fraction; $LVID_d$, left ventricle (LV) end-diastolic inner diameter; $LVID_s$, LV end-systolic inner diameter; ND, normal diet; HFD, high fat diet. Data are mean±SD, n=6-8, *$P<0.05$ vs. Control-ND, #$P<0.05$ vs. Control-HFD and †$P<0.05$ vs. Tg-ABAT/tTA-ND.
